# Comparative Chemistry of *Aspergillus oryzae* (RIB40) and *A. flavus* (NRRL 3357)

**DOI:** 10.3390/metabo2010039

**Published:** 2012-01-05

**Authors:** Christian Rank, Marie Louise Klejnstrup, Lene Maj Petersen, Sara Kildgaard, Jens Christian Frisvad, Charlotte Held Gotfredsen, Thomas Ostenfeld Larsen

**Affiliations:** 1 Department of Systems Biology, Center for Microbial Biotechnology, Technical University of Denmark, Søltofts Plads B221, DK-2800 Kgs. Lyngby, Denmark; Email: christian.rank@gmail.com (C.R.); marlk@bio.dtu.dk (M.L.K.); lmape@bio.dtu.dk (L.M.P.); sarakildgaard@hotmail.com (S.K.); jcf@bio.dtu.dk (J.C.F.); 2 Department of Chemistry, Technical University of Denmark, Kemitorvet B201, DK-2800 Kgs. Lyngby, Denmark; Email: chg@kemi.dtu.dk

**Keywords:** *Aspergillus oryzae*, (RIB40), *Aspergillus flavus*, (NRRL 3357), parasiticolide, ditryptoleucine, oryzamide

## Abstract

*Aspergillus oryzae* and *A. flavus* are important species in industrial biotechnology and food safety and have been some of the first aspergilli to be fully genome sequenced. Bioinformatic analysis has revealed 99.5% gene homology between the two species pointing towards a large coherence in the secondary metabolite production. In this study we report on the first comparison of secondary metabolite production between the full genome sequenced strains of *A. oryzae* (RIB40) and *A. flavus* (NRRL 3357). Surprisingly, the overall chemical profiles of the two strains were mostly very different across 15 growth conditions. Contrary to previous studies we found the aflatrem precursor 13-desoxypaxilline to be a major metabolite from *A. oryzae* under certain growth conditions. For the first time, we additionally report *A. oryzae* to produce parasiticolide A and two new analogues hereof, along with four new alkaloids related to the *A. flavus* metabolites ditryptophenalines and miyakamides. Generally the secondary metabolite capability of *A. oryzae* presents several novel end products likely to result from the domestication process from *A. flavus*.

## 1. Introduction

*Aspergillus oryzae* is one of industry’s most used “workhorses” and has been used for centuries in food fermentation for the production of e.g., sake, soy sauce and other traditional Asian foods [1]. *A. oryzae* is also a widely used organism for production of amylase, lipases and proteases and more recently also for heterologous expression of secondary metabolite genes and non-fungal proteins [2,3,4]. For many years, *A. oryzae* has been suspected to be a domesticated form of *A. flavus*, a plant and mammalian pathogenic saprophyte, capable of producing some of the most carcinogenic compounds known: the aflatoxins. Genetic work and subsequent genome sequencing of strains of both species have verified the tight link between the species [1,5,6,7,8].

**Figure 1:**
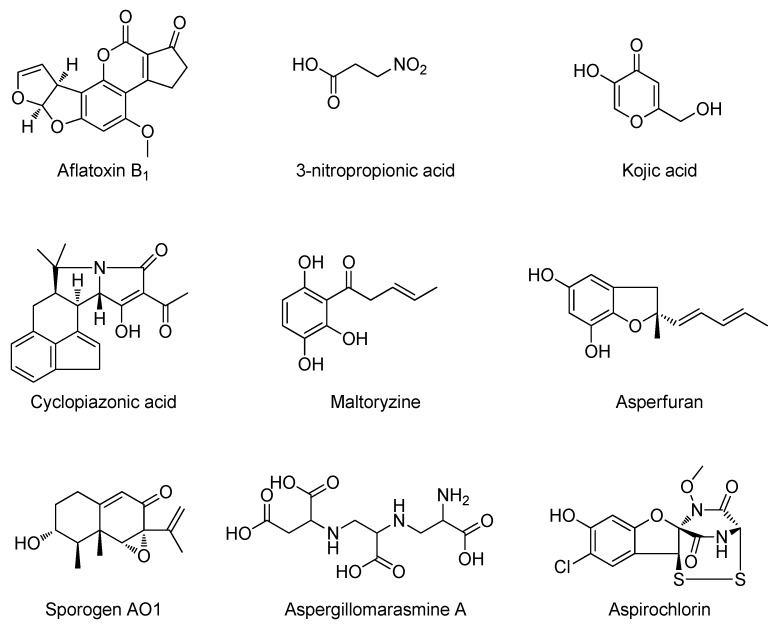
Known compounds from *Aspergillus flavus* or *A. oryzae.*

The relationship of the two species has resulted in extensive screening of the toxic potential of *A. oryzae*, but no genuine evidence of aflatoxin production in validated *A. oryzae* isolates has ever been shown. Other important toxins, known from *A. flavus*, have on the other hand been shown in *A. oryzae*: 3-Nitropropionic acid [9] and cyclopiazonic acid (CPA) [10] along with kojic acid [11,12] (Figure 1). Additional metabolites previously reported from *A. oryzae* are asperfuran [13], sporogen AO1 [14,15], maltoryzine [16], and aspergillomarasmine A [17,18]. Aspirochlorine has been found in *A. flavus*, *A. oryzae* and *A. tamarii* [19,20,21,22,23] (Figure 1). For reviews on the safety and taxonomy of *A. oryzae*, see [7,24,25,26].

The few predicted differences between the genomes of *A. oryzae* and *A. flavus* (ca. 99.5% genome homology and 98% at the protein level for RIB40/ATCC 42149 and NRRL 3357 [27]), could lead one to expect *A. oryzae* to produce most of the metabolites found in *A. flavus* [1,28,29,30,31], but published metabolic data indicates a very low chemical correlation [32]. It is with reference to the established genetic heritage of *A. oryzae* from *A. flavus* remarkable that maltoryzine, sporogen AO1, asperfuran and aspergillomarasmine A have never been unambigously identified in *A. flavus*. Though research on *A. flavus* chemistry has focused primarily on toxic compounds, one would expect that these metabolites should be part of its chemical potential as they are for the domesticated *A. oryzae*. The preliminary bioinformatic studies in conjunction with the genome sequencing shows roughly the same number of predicted genes: 32 Polyketide synthases (PKSs) and 28 non-ribosomal synthases (NRPSs) for *A. flavus* and 32 PKSs and 27 NRPSs for *A. oryzae* with 2 NRPSs apparently unique for each strain [33]. The exclusiveness of these genes in terms of end product has yet to be verified chemically.

Most of the predicted genes for secondary metabolites of *A. oryzae* (or *A. flavus*) have not been mapped to specific metabolic products, despite the genome sequencing of RIB40 in 2005 [27]. Only genes of the most important toxins: Aflatoxin [31,34,35], CPA [36,37] and aflatrem [38] have been fully annotated in both species, which leaves much to be explored. The full chemical potential of either species is unknown and epigenetic modifiers [39,40,41,42,43] may be necessary, alongside with the use of different growth conditions to aid triggering the full potential of secondary metabolite expression in these two closely related species.

The aim of the current work has been to perform an initial comparative investigation of the chemistry from the two genome sequenced strains of *A. oryzae* (RIB40) and *A. flavus* (NRRL 3357), in order to get further insights into possible homologies and differences in secondary metabolite production for these two important species.

## 2. Results and Discussion

### 2.1. De-Replication of A. oryzae RIB40

For the analysis of *A. oryzae* RIB40 chemistry, we investigated a series of solid media (YES, YESBEE, DRYES, CYA, CYAS, CY20, CY40, DUL, GAK, GMMS, MEA, OAT, PDA, TGY, WATM (see Methods and Materials for explanation) cultivations with micro-scale extractions [44,45] and subsequently analyzed with HPLC-DAD-MS for selection of optimal conditions. The different media were tested on a collection of *A. oryzae* (RIB40, IBT 28103) and *A. flavus* (NRRL 3357, IBT 23106, IBT 3642) and these strains were cultivated at 25 °C in the dark for 7 and 14 days. The media screening for *A. oryzae* and *A. flavus* indicated the greatest chemodiversity and metabolite production from CYA, YES and WATM agar for our purpose.

**Figure 2:**
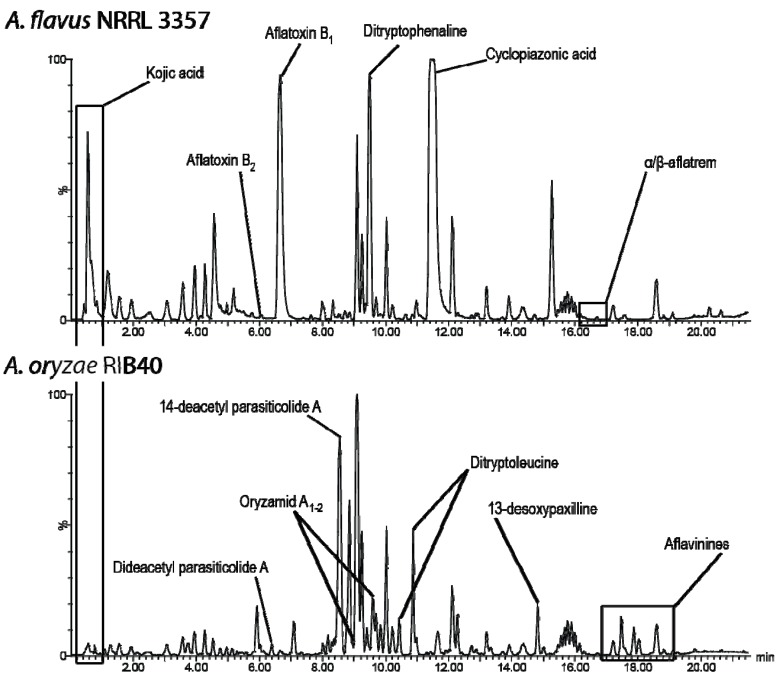
ESI+ BPC chromatogram of 7 day micro scale extract from WATM, bottom: *A. oryzae* RIB40, top: *A. flavus* NRRL 3357. Besides kojic acid (shown in box) and analogues in the beginning of chromatogram and ergosterol in the end (not shown), there are very few identical compounds between the genetically almost identical strains. Note that aspirochlorine is only detectable in negative ionization, and therefore not visible in this chromatogram.

The comparison of the secondary metabolite profiles of the two strains, NRRL 3357 and RIB40, exposed a surprisingly high degree of chemical difference on all media as illustrated in Figure 2 and Table 1 for the WATM medium. The major metabolite repetitions between the two genome sequenced strains were merely kojic acid and ergosterol, like a number of minor metabolites (not analyzed here) seemed to be shared between the two strains. Altogether, this is in sharp contrast to the high gene homology, particularly for the secondary metabolite genes.

**Table 1:** LC-MS de-replication of some of the important secondary metabolite pathways from the two full genome sequenced siblings, *A. flavus* (NRRL 3357) and *A. oryzae* (RIB40). Based on 7 day fermentation on solid WATM agar in the dark. (+) indicates the presence of these types of compounds in *A. flavus* based on UV spectroscopic analysis. * New compounds reported here for the first time.

Metabolite	*A. flavus* NRRL 3357	*A. oryzae* RIB40
Kojic acid	+	+
Aflatoxin	+	-
Aflavinines	(+)	+
Aflatrem	+	13-desoxypaxilline
Miyakamides	(+)	Oryzamides *
Aspirochlorine	-	+
Cyclopiazonic acid	+	-
Ditryptophenaline	+	Ditryptoleucine *
Parasiticolide A	-	14-deacetyl parasiticolide A *

Known metabolites to *A. oryzae* were de-replicated and we found that the RIB40 strain did not produce detectable levels (LC-MS) of CPA (as also noted by Tokuoka *et al.* [36]), asperfuran, sporogen AO1, maltoryzine or aspergillomarasmine under these growth conditions. It did, however, produce kojic acid and aspirochlorine and a series of potentially new metabolites of which some were isolated, structurally characterized and reported here.

### 2.2. New Metabolites to A. oryzae RIB40

During fermentation of the chemically potent RIB40 strain, we have been interested in the tremorgenic compounds, allegedly coupled to fungal sclerotia [46,47,48,49,50,51,52,53,54] and whether these could be found in *A. oryzae* as they have been in *A. flavus*. The RIB40 strain produces large and abundant sclerotia, especially on WATM agar, a fact not widely announced in literature although sclerotia have been observed in *A. oryzae* sporadically [55,56,57]. No sclerotia were observable after 14 days on YES agar, but although these metabolites are often characterized as sclerotial metabolites, there is not a strict correlation between the biosynthesis of these metabolites and the formation of sclerotia, as also noted by Wilson [58], and this extract was used for the described isolations.

**Figure 3:**
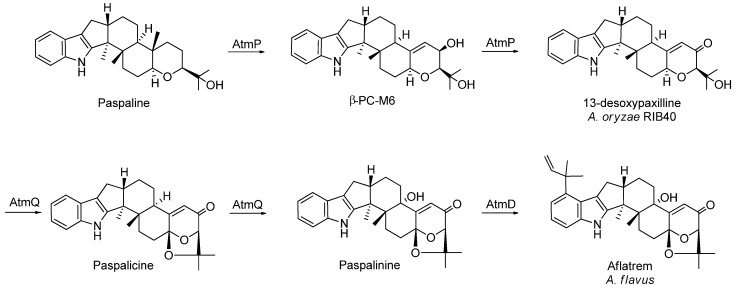
The final steps in the proposed biosynthesis of aflatrem (in *A. flavus*). *A. oryzae* RIB40 biosynthesis stops at 13-desoxypaxilline [38].

Here, we report the discovery of the aflatrem precursor 13-desoxypaxilline (13-dehydroxypaxilline) in *A. oryzae* RIB40, originally isolated from *Penicillium paxilli* [59,60,61,62,63]. Aflatrem is known from *A. flavus* and was discovered by Wilson and Wilson in 1964 [64] and structure elucidated by Gallagher *et al.* in 1978 and 1980 [65,66]. 13-desoxypaxilline was present in YES, CYA, OAT and WATM agar 7 day old micro-scale extracts. From the 14 days old YES 200 plate extract used for isolation, 13-desoxypaxilline was recovered as an intermediate metabolite. LC-MS, LC-MS/MS and NMR data analysis (Supplementary Material) confirmed the structure. Naturally the prospect of finding aflatrem itself was investigated, though no apparent peak was visible in HPLC-DAD data files. The use of a LC-MS/MS method further confirmed 13-desoxypaxilline as an end-product of *A. oryzae* RIB40 for the above cultivation conditions, since none of the proposed intermediate steps towards aflatrem could be detected (LC-MS/MS) and only one sample (WATM, 7d) showed traces of paspaline, a precursor for 13-desoxypaxilline (Figure 3).

A second extract was made from 100 plates of a 14-day old *A. oryzae* RIB40 culture grown on WATM agar with abundant sclerotia formation to validate the findings from the YES extract. The analysis of the WATM extract showed 13-desoxypaxilline as a major metabolite alongside other sclerotium-related metabolites, such as aflavinines (based on UV-data not shown). The discovery of 13-desoxypaxilline as the apparent end-product of *A. oryzae* RIB40 is in agreement with the analysis of Nicholson *et al.* [38], who showed that a frameshift mutation in the *atmQ* gene presumably accounts for 13-desoxypaxilline not being converted into paspalicine and paspalinine. This mutation is therefore likely responsible for terminating the aflatrem biosynthesis in RIB40 prematurely, short of the acetal ring closure, C-13 hydroxylation and isoprene attachment. Nicholson *et al.* [38] demonstrated that AtmQ is a multifunctional cytochrome P450 monooxygenase likely to catalyze the several oxidative steps needed for biosynthesis of the acetal ring present in the structures of paspalicine, paspalinine and aflatrem, altogether pointing towards a more complex biosynthesis than shown here. Contrary to our discovery, Nicholson *et al.* did not find the aflatrem gene cluster of RIB40 to be transcribed during their fermentations [38].

The isolated 13-desoxypaxilline is a member of the paspalitrem tremorgens, a widely distributed group of metabolites that have been isolated from several genera: *Penicillium*, *Eupenicillium*, *Claviceps*, *Emericella*, *Aspergillus* and *Phomopsis* [67,68,69]. Besides the tremorgenic activity in animals, these metabolites have been shown to be insecticides [68], which is believed to be their ecological function together with aflatoxin and CPA for protection of the sclerotia against fungivorous insects [46,47].

**Figure 4:**
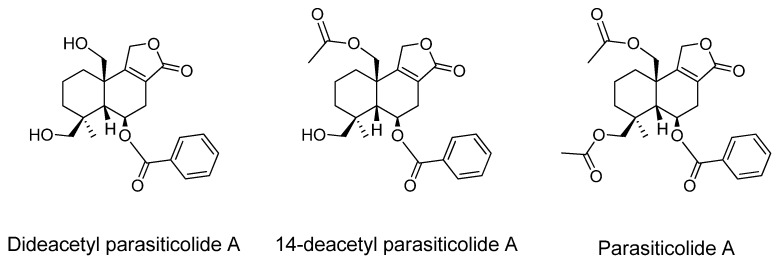
Structures of dide- and 14-deacetyl parasiticolide A and parasiticolide A.

In addition to 13-desoxypaxilline, two new analogues of parasiticolide A were also isolated and are here reported for the first time. The metabolites showed to be dide- and 14-deacetoxy analogues, and are most likely precursors to the sesquiterpene parasiticolide A (= astellolide A) (see Figure 4). The metabolites were present in CYA, YES and WATM extracts and isolated from the same 14 day old YES extract as 13-desoxypaxilline, and the dide- and 14-deacetyl analogues were also found in the sclerotia enriched WATM extract. Again the metabolites were analyzed using LC-MS and NMR. Several different extraction procedures were tested to verify the correctness of the compounds as genuine metabolites and not as *in vitro* degraded parasiticolide A products, but all samples showed only dide- and 14-deacetyl parasiticolide A and no traceable (LC-MS) levels of parasiticolide A itself, even with different non-acidic extractions. Parasiticolide A have been isolated from *A. flavus* var. *columnaris* (FKI-0739) once [71] and was originally isolated and characterized from *A. parasiticus* (IFO 4082) [72,73] and later also from a mutant of *Emericella variecolor* (= *A. stellatus* Curzi) [74]. Recently parasiticolides have been detected in the newly described species *A. arachidicola* (CBS 117610) and *A. minisclerotigenes* (CBS 117635) [75]. There have to our knowledge not been published any toxic studies on the parasiticolides, but the related peniopholides from the fungus *Peniophora polygonia* have been reported to have antifungal properties [76].

In our observations parasiticolides are more often detectable metabolites of *A. oryzae* than of *A. flavus* under the same fermentation conditions, suggesting that the pathway is partly silenced for *A. flavus* and may need epigenetic modification to be expressed under otherwise normal growth conditions. It is interesting that parasiticolide A is scarcely observed in *A. flavus*, when it is an important product of *A. oryzae* and also of *A. parasiticus*. As for 13-desoxypaxilline, dide- and 14-deacetyl parasiticolide A are almost certainly products of a prematurely ended biosynthesis, here parasiticolide A. We also isolated and elucidated a third parasiticolide A analogue; a formyl variant of parasiticolide A, but it was not possible to exclude the possibility of in vitro chemistry due to the formic acid added during the ethyl acetate extraction, so the correctness of this metabolite remains tentative. (Hamasaki *et al.* used benzene to extract parasiticolide A [77]). All paraciticolide analogues were structure elucidated by NMR and shift values correlated with the published data for parasiticolide A, except for the missing signals of the acetate units and their minor influence on the chemical shifts values of adjacent protons and carbons (See Supplementary Material for NMR data).

To further verify these observations, a MS/MS method was used to analyze several different microscale extracts of RIB40 for parasiticolide A itself. Trace amounts of parasiticolide A was found under these conditions and compared to an isolate of *A. parasiticus* (IBT 4387) capable of producing parasiticolide A. In the *A. parasiticus* isolate no dide- or 14-deacetyl parasiticolide A could be measured, indicating a complete transformation into the end-product (Figure 4). The small amount of parasiticolide A in RIB40 (roughly 1:1.000 ratio compared to 14-acetyl parasiticolide A, presuming the same response factor) might be the result of spontaneous acetylation involving the first acetylating enzyme. When the gene cluster of this metabolite is mapped, it is likely that the gene responsible for the last (specific) acetylation will be found to be mutated. Except for the section *Nidulantes* member *Emericella variecolor*, all other producers of these metabolites have been members of section *Flavi*. No indication points to these metabolites being part of the previously mentioned sclerotium metabolites since they are not found in selective sclerotium extracts, but are found for example in *A. arachidicola*, which is not known to produce sclerotia.

**Figure 5:**
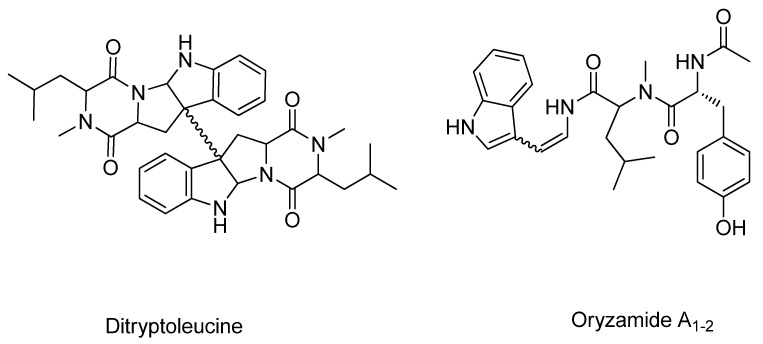
Structure of the new *A. oryzae* metabolites: Ditryptoleucine and oryzamide A_1-2_.

Four new *A. flavus* related NRPS compounds were also isolated from *A. oryzae* RIB40 and characterized based on standard interpretation of ^1^H, COSY, HMQC and HMBC NMR data (see Supplementary Material). The compounds showed to be two isomeric metabolites named ditryptoleucine related to the ditryptophenalines [78,79,80,81] and the two indole-enamides named oryzamides A_1-2_ (*cis*- and *trans*-forms) structurally similar to the antibiotic miyakamide B_1-2_ [71] (Figure 5).

It proved difficult to unambiguously isolate the *cis*- and *trans*-isomer of oryzamide A in a pure form due to isomerization around the double bond. Due to this the structure elucidation has been performed on mixtures of compounds. It is evident from the structure elucidation that oryzamides A_1-2_ only differ in the configuration around the double bond of the enamine as evident from the size of the coupling constant. Marfey analysis of the amino acid derived compounds only established the presence of L-tyrosine in the oryzamides. NMR structural data on the trans isomer (A_1_) is present in the supporting material.

Intriguingly, these *A. oryzae* metabolites have apparently exchanged phenylalanine with leucine compared to the similar *A. flavus* metabolites, indicating either a common trait in the domestication process or a coupling between the two pathways. The diketopiperazine ditryptoleucine was isolated in two variants with ^1^H-NMR shifts varying around the C-C dimeric bond, but with the same base structure. This unspecific dimerization is in line with previously isolated compounds. A hybrid between the ditryptophenaline and ditryptoleucine was isolated from an *Aspergillus* sp. as WIN 64745, with both a phenylalanine and leucine moiety, but with no *N*-methylation [79]. These compounds were tested and proved antagonistic against substance P at the NK1 receptor [82]. The oryzamides A_1-2_ are variants of the miyakamides B_1-2_ [71]. The exchanged phenylalanine has been substituted with leucine between a tryptophan and a tyrosine in the oryzamides. However this exchange of phenylalanine and leucine has often been found for compounds produced within the same species. *Penicillium polonicum* (= *P. fructigenum*) [83,84,85] produces both fructigenine A = puberuline = rugulosuvine A, containing phenylalanine and fructigenine B = verrucofortine, containing leucine. We could also detect two slightly later eluting compounds in the *A. oryzae* RIB40 extracts likely to be two further indole-enamides having phenylalanine incorporated instead of tyrosine as evident from LC-DAD-MS analysis (data not shown) as also seen for the miyakamides [71].

## 3. Experimental

General procedures and methods for analysis are described in [43,44,86].

Mass spectrometric analysis was performed using a Agilent HP 1100 liquid chromatograph with a DAD system (Waldbronn, Germany) on a LCT oaTOF mass spectrometer (Micromass, Manchester, UK) with a Z-spray ESI source and a LockSpray probe. For general procedures see [87]; method 1 for LC-DAD-TOF was used in this study. All solvents used were HPLC grade from Sigma-Aldrich (St. Louis, MO, USA).

### 3.1. Fungal Material and Fermentation

*A. oryzae* RIB40 (IBT28103); *A. flavus* NRRL3357 (IBT3696), (IBT15934), NRRL 13462; *A. parvisclerotigenus* IBT16807 and *A. minisclerotigenes* IBT13353 were obtained from the IBT Culture Collection at DTU Systems Biology, Technical University of Denmark. The RIB40 isolate used for isolation of 13-dehydroxypaxilline was cultured for 14 days at 25 °C in the dark on 200 Petri dishes with Yeast Extract Sucrose agar (YES). All strains were grown for 1 week at 25 °C on YES, Czapek Yeast Autolysate (CYA), Wickerhams Antibiotic Test Medium (WATM) agar [88], YESBEE [89] (YES+50 g Bee pollen Type III, granulate, Sigma, P-8753, pr. 1 L medium), DRYES (Dichloran rose Bengal chloramphenicol agar), AFPA (*Aspergillus flavus*, *A. parasiticus* agar), CYAS (CYA + 50 g NaCl pr. 1 L medium), CY20 (CYA + 170 g sucrose pr. 1 L), CY40 (CYA with added 370 g sucrose pr. 1 L medium), DUL (Dulaney’s medium for Penicillin), GAK (Potato-carrot agar), GMMS (Glucose minimal media (GMM) + 2% sorbitol), MEA (Malt extract agar), OAT (Oat meal agar), PDA (Potato-dextrose agar). For medium formulations see Samson *et al.* [90].

### 3.2. Extraction and Isolation of Pure Compounds

*13-Desoxypaxilline*. The plates were homogenized using a Stomacher and 100 mL EtOAc with 1% HCO_2_H pr. 10 plates. The extract was filtered and dried down on a freeze drier. The crude extract was separated on a KP-C18-HS 60 g SNAP column using a Biotage Isolera One (Biotage, Uppsala, Sweden), resulting in a 22 mg fraction. The fraction was segmented with a 10 g ISOL Diol column, using 12 steps of stepwise Heptane-dichloromethane-EtOAc-MeOH. 13-desoxypaxilline was predominant in a 100% EtOAc fraction (6 mg), and purified on a Waters HPLC W600/996PDA (Milford, MA, USA) and a RP column (Phenomenex Luna C18(2), 250 × 10 mm, 5 µm, Torrance, CA, USA) using a gradient of 80% MeCN (H_2_O–Milli-Q (Millipore, MA, USA)) to 90% over 10 min. with 50 ppm TFA added to the solvents. The collection was concentrated on a rotary evaporator (Büchi V-855/R-215, Flawil, Switzerland) and dried under N_2_ to yield 0.5 mg of white, amorphous 13-desoxypaxilline.

*Dideacetyl-, 14-deacetyl-, and 18-formyl parasiticolide A*. From the same fermentation described for 13-desoxypaxilline a more polar, 90 mg fraction was fractionated with a 10 g ISOL Diol column, using 12 steps of stepwise Heptane-dichloromethane-EtOAc-MeOH. The parasiticolide A-analogues were predominant in a 100% EtOAc fraction (10 mg), and purified on a Waters HPLC W600/996PDA (Milford, MA, USA) and a RP column (Phenomenex Luna C18(2), 250 × 10 mm, 5 µm, Torrance, CA, USA) using a gradient of 72% MeCN (H_2_O–Milli-Q (Millipore, MA, USA)) to 87% over 15 min. with 50 ppm TFA. The collection was concentrated on a rotary evaporator (Büchi V-855/R-215) and dried under N_2_ to yield 0.3, 1.0 and 0.8 mg of white, amorphous di-, 14-deactyl- and 18-formyl parasiticolide A, respectively. See Supplementary Material for NMR data.

*Ditryptoleucine.* 400 plates of *A. oryzae* RIB40 were cultured on YES medium. The plates were homogenized using a Stomacher and 100 mL EtOAc pr. 10 plates. The extract was filtered and dried down on a freeze drier. The crude extract were separated into three phases by dissolving it in 9:1 MeOH:H_2_O–Milli-Q and extracted into a heptan phase and afterwards a DCM phase. The DCM phase was separated on a KP-C18-HS SNAP column using a Biotage Isolera One (Biotage, Uppsala, Sweden) using a gradient of 10% MeOH (H_2_O–Milli-Q (Millipore, MA, USA)) to 100% over 15 CVs (column volumes) resulting in a 402 mg fraction. The fraction was further separated on another KP-C18-HS SNAP column using a Biotage Isolera One (Biotage, Uppsala, Sweden) using a gradient of 10% MeOH (H_2_O–Milli-Q (Millipore, Ma, USA)) to 25% over 2 CVs, 25% MeOH to 50% over 8 CVs and 50% to 100% over 4 CVs resulting in a 60.1 mg fraction. The fraction was fractionated on a LH-20 column using 100% MeOH. The ditryptoleucines were present in the earlier fractions and were purified on a Waters HPLC W600/996PDA (Milford, MA, USA) using a RP column (Phenomenex Luna C18(2), 250 × 10 mm, 5 µm, Torrance, CA, USA) using a gradient of 50% MeCN (H_2_O–Milli-Q (Millipore, MA, USA)) to 100% over 20 min. with 50 ppm TFA and a flow of 4 mL/min. The collections were concentrated on a rotary evaporator (Büchi V-855/R-215) and dried under N_2_ to yield 4.0 mg of the two isomers. See Supplementary Material for NMR data.

*Oryzamides A_1-2_.* The remaining broth from the fermentation described for ditryptoleucine was extracted with EtOAc + 1% HCO_2_H for 24 h. The extract was filtered and dried down on a freeze drier. The crude extract were separated into three phases by dissolving it in 9:1 MeOH:H_2_O–Milli-Q and extracted into a heptan phase and afterwards a DCM phase. The DCM phase was separated on a KP-C18-HS SNAP column using a Biotage Isolera One (Biotage, Uppsala, Sweden) using a gradient of 10% MeOH (H_2_O–Milli-Q (Millipore, MA, USA)) to 100% over 15 CVs (column volumes) resulting in a 654 mg fraction. The fraction was fractionated with a 10 g ISOL Diol column, using 13 steps of stepwise Hexane-dichloromethane-EtOAc-MeOH. The miyakamides were predominant in the 40:60 DCM:EtOAc and 20:80 DCM:EtOAc fractions (43.8 mg), and purified on a Waters HPLC W600/996PDA (Milford, MA, USA) and a RP column (Phenomenex Luna C18(2), 250 × 10 mm, 5µm, Torrance, CA, USA) using a gradient of 45% MeCN (H_2_O–Milli-Q (Millipore, MA, USA)) to 62% over 15 min. with 50 ppm TFA. The collection was concentrated on a rotarvap (Büchi V-855/R-215) and dried down under N_2_ to yield 4.0 and 2.0 mg of oryzamide A_1_ and A_2_, respectively. See Supplementary Material for NMR data.

### 3.3. Marfeys Method

100 µg of each peptide was hydrolysed with 200 µL 6 M HCl at 110 °C for 20 h. To the hydrolysis products (or 50 µL (2.5 µmol) solutions of standard D- and L-amino acids) was added 50 µL water, 20 µL 1 M NaHCO_3_ solution and 100 µL 1% FDAA in acetone, followed by reaction at 40 °C for 1 h. The reaction mixture was removed from the heat, neutralized with 10 µL 2 M HCl and the solution was diluted with 820 µL MeOH to a total volume of 1 mL. The FDAA derivatives were analysed by UHPLC-DAD on a Dionex Ultimate 3000 RS DAD equipped with a Kinetex C18 column (2.6 μm, 150 × 2.10mm, Phenomenex). The analyses were run with a gradient elution of MeCN/H_2_O–Mili-Q (Millipore, MA, USA) added 50 ppm TFA from 15 to 100% MeCN over 7 min (60 °C, 0.8 mL/min). The retention times of the FDAA derivatives were compared to retention times of the standard amino acid derivatives.

### 3.4. Selective Extraction of Sclerotium Metabolites

The selective extraction of sclerotia from IBT 15934, NRRL 13462, IBT 16807 and IBT 13353 was made from harvested sclerotia of a 7 day old cultivation on WATM and CYA agar (25 °C in dark). The sclerotia were washed several times with Milli-Q (Millipore, Millford, USA) 0.22 µm H_2_O and dried. The sclerotia were transferred to a 2 mL Eppendorf tube together with three stainless steel balls (2 × 1 mm and 1 × 5 mm) and frozen with liquid N_2_ before mechanical crushed. The pulverized sclerotia were suspended in 1mL methanol and transferred to a 2 mL vial with 1 mL of 1:2:3 methanol:dichloromethane:ethylacetate and left for evaporation overnight in a fume hood. The dried extract was resolved in 1 mL methanol in ultrasonicated for 10 min and then filtered with a 0.45 µm PTFE filter to a clean vial for analysis.

### 3.5. HPLC-DAD-TOF Method

Mass was measured using a Agilent HP 1100 liquid chromatograph with a DAD system (Waldbronn, Germany) on a LCT oaTOF mass spectrometer (Micromass, Manchester, UK) with a Z-spray ESI source and a LockSpray probe. For general procedures see [86], method 1.

### 3.6. NMR Instrumentation

NMR spectra were recorded on a Varian Unity Inova 500 MHz spectrometer equipped with a 5 mm probe using standard pulse sequences. The signals of the residual solvent protons and solvent carbons were used as internal references (*δ*_H_ 2.50 and *δ*_C_ 39.5 ppm for DMSO). In cases where higher field was needed the NMR spectra were recorded on a Bruker Avance 800-MHz spectrometer equipped with a 5-mm TCI Cryoprobe at the Danish Instrument Center for NMR Spectroscopy of Biological Macromolecules.

### 3.7. MS/MS Method Used for Aflatrem and Parasiticolide Screening

Liquid chromatography was performed on an Agilent (Torrence, CA, USA) 1100 HPLC system coupled to a triple-quadropole mass spectrometer (Waters-Micromass, Manchester, UK) with a Z-spray ESI operated in positive mode source using a flow of 700 L/h nitrogen desolvated at 350 °C. The hexapole was held at 50 V. The system was controlled by MassLynx v4.1 (Waters-Micromass). Nitrogen was used as collision gas, and the MS operated in MRM mode (dwell time = 100 ms) with the parameters listed in Table 2. Extracts of 2 µL were injected and separated on a Phenomenex Gemini C_6_-phenyl, 3 µm, 2 × 50 mm column with a flow of 0.3 µL/min. Water contained 20 mM ammonium formate. Oven temperature 40 °C. Two different methods were applied to the aflatrem- and parasiticolide screen: Aflatrem inlet method: Linearly gradient from 50 to 100% MeCN over 5 min, increased to 0.5 µL/min over 1.5 min. The column was washed additionally 1.5 min with 100% MeCN at 0.5 µL/min, followed by a return to 50% MeCN over 2.5 min and kept at this level for another 1 min with a linearly decrease in flow to 0.3 µL/min, prior to the next sample. Standards used for analysis of this pathway were from Sigma-Aldrich Aldrich (St. Louis, MO, USA). Parasiticolides: linearly gradient from 20 to 90% MeCN over 15 min, increased to 0.5µL/min from 90 to 100% MeCN in additional 1 min. The column was washed from 2 min with 100% MeCN at 0.5 µL/min, followed by a return to 20% MeCN over 1.5 min and kept at this level for another 3.5 min with a linearly decrease in flow to 0.3 µL/min, before the next sample. Standards were internal standards from other extracts of the known parasiticolide A producer *A. parasiticus*: IBT 4387 (= CBS 260.67) and IBT 11863 (= CBS 115.37).

**Table 2:** MS/MS method including scan event, retention times, transition ions and the cone and collision energies used.

Compound	Scan event	RT(min)	Ion type	Transition ( *m*/*z*) ^a^	Cone (V)	Collision energy (eV)
Paxilline	1	4.0	Quantifier	436 → 130	2525	3030
			Qualifier	436 → 182		
Paspalinine	2	4.3	Quantifier	434 → 130	2525	2020
			Qualifier	434 → 376		
13-desoxypaxilline	3	4.8	Quantifier	420 → 182	2525	3030
			Qualifier	420 → 130		
Aflatrem	4	5.2	Quantifier	502 → 198	2525	2020
			Qualifier	502 → 445		
Paspaline	5	5.5	Quantifier	422 → 130	2525	2020
			Qualifier	422 → 275		
Dideacetyl-parasiticolide A	1	7.0	Quantifier	387 → 217	3030	4040
			Qualifier	387 → 189		
14-deacetyl parasiticolide A	2	8.7	Quantifier	429 → 217	3030	4040
			Qualifier	429 → 189		
Parasiticolide A	3	10.4	Quantifier	488 → 229	3030	3030
			Qualifier	488 → 247		

^a^ All transitions were made from [M+H]^+^, except for paraciticolide A: [M+NH_4_]^+^.

## 4. Conclusions

The tremorgenic 13-desoxypaxilline has been isolated from *A. oryzae* RIB40 and verified under several different growth conditions contrary to previous studies. We believe that 13-desoxypaxilline is the end-product of the aflatrem biosynthesis for the RIB40 strain since no aflatrem could be detected in any fermentation using LC-MS/MS as detection.

The new metabolites dide- and 14-deacetyl parasiticolide A were also found as genuine products from the RIB40 strain and the compounds were present in multiple fermentations, however parasiticolide A was only detected in trace amounts using a LC-MS/MS method. This indicates a defective acetylation of the 14-deacetyl parasiticolide A and the small amount of parasiticolide A in RIB40 could be the result of spontaneous acetylation in the cell cytosol. The mono-deacetylated analogue detected in both *A. flavus* and *A. oryzae* had same retention times, suggesting a selective acetylation.

The new NRPS compounds ditryptoleucine and oryzamides A_1-2_ appear to be natural variants of known *A. flavus* metabolites. They share the exchange of a phenylalanine for a leucine, although they are believed to originate from two unrelated pathways.

Altogether, our findings contribute to understanding why the overall chemical profiles of *A. oryzae* (RIB40) and *A. flavus* (NRRL 3357) appear quite different since some of the end-products usually seen in *A. flavus* are apparently not reached in *A. oryzae*. Whether the different chemical profiles are merely the result of different regulation that can be overcome by the use of epigenetic modifiers or are a result of genuine mutations remains to be settled. *A. oryzae* RIB40 is clearly a chemically potent strain, and as more of its chemistry is unfolded we believe that most of the biosynthetic pathways of *A. flavus* will be found to be more or less functional. Future research will reveal whether the many different *A. oryzae* strains that are used as industrial workhorses are as chemically potent as the RIB40 strain.

## Supplementary Files

Supplementary File 1 DOCX-Document (DOCX, 110 KB)
