# Supplementary material for: Comparative Chemistry of Aspergillus oryzae (RIB40) and A. flavus (NRRL 3357)
Source: Metabolites. 2012 Jan 5;2(1):39–56. doi: 10.3390/metabo2010039 (PMC3901201; doi:10.3390/metabo2010039)
Supplement: Supplementary File 1 — DOCX-Document (DOCX, 110 KB) [file metabolites-02-00039-s001.docx]

**Supplementary Material**

Comparative chemistry of *Aspergillus oryzae* (RIB40) and  *A. flavus* (NRRL 3357)

Christian Rank ^1,†^, Marie Louise Klejnstrup ^1,†^, Lene Maj Petersen ^1^, Sara Kildgaard ^1^,
Jens Christian Frisvad ^1^, Charlotte Held Gotfredsen ^2^ and Thomas Ostenfeld Larsen ^1,^*

13-Desoxypaxilline

HRESIMS: *m/z* = 420.2551 [M+H]^+^, calculated for [C_27_H_33_NO_3_+H]^+^: 420.2533.

NMR spectra were acquired in DMSO-*d_6_* on a Bruker Avance 800 MHz spectrometer using standard pulse sequences. ^1^H-NMR (799.30 MHz, DMSO-*d_6_*, 25 °C, 2.50 ppm): 0.88 (3H, s, H-23), 1.00 (3H, s, H-25), 1.16 (3H, s, H-29), 1.20 (3H, s, H-28), 1.52 (1H, ddd, *J* = 25.4, 12.8, 4.4 Hz, H-14a), 1.60 (1H, m, H-14b), 1.65 (1H, m, H-15a), 1.74 (1H, d, *J* = 12.2 Hz, H-15b), 1.81 (1H, ddd, *J* = 17.9, 13.8, 4.2 Hz, H-6a), 1.98 (1H, ddd, *J* = 13.8, 13.6, 4.2 Hz, H-5a), 2.07 (1H, m, H-5b), 2.22 (1H, m, H-6b), 2.32 (1H, dd, *J* = 12.8, 11.0 Hz, H-17a), 2.53 (1H, m, H-13), 2.62 (1H, dd, *J* = 12.8, 6.3 Hz, H-17b), 2.71 (1H, m, H-16), 3.74 (1H, d, *J* = 1.6 Hz, H-9), 4.34 (1H, br. s, 27-OH), 4.41 (1H, m, H-7), 5.73 (1H, s, H-11), 6.91 (1H, dd, *J* = 7.6, 7.6 Hz, H-21), 6.95 (1H, dd, *J* = 7.6, 7.6 Hz, H-22), 7.27 (1H, d, *J* = 7.6 Hz,
H-23), 7.28 (1H, d, *J* = 7.6 Hz, H-20), 10.76 (1H, s, N-H).

^13^C-NMR (201.00, DMSO-*d_6_*, 25 °C, 39.5 ppm): 14.4 (C-25), 15.4 (C-26), 23.5 (C-15), 24.8 (C-14), 25.5 (C-28), 25.7 (C-29), 26.7 (C-17), 29.5 (C-6), 30.7 (C-5), 41.6 (C-13), 48.5 (C-16), 49.2 (C-3), 49.8 (C-4), 70.7 (C-27), 74.0 (C-7), 82.4 (C-9), 111.6 (C-23), 115.8 (C-18), 117.5 (C-20), 118.1 (C-21), 119.2 (C-22), 120.7 (C-11), 124.4 (C-19), 140.2 (C-24), 150.4 (C-2), 168.6 (C-12), 196.1 (C-10).

14-Deacetyl parasiticolide A

All NMR spectra of parasiticolide A analogues were acquired in DMSO-*d*_6_ on a Varian Unity Inova 500 MHz spectrometer with 4 mm gHX Nano probe and with a spin rate of 2 kHz for all samples, using standard pulse sequences. The spectra were referenced to this solvent with resonances δ_H_ = 2.50 and δ_C_ = 39.5.

HRESIMS: *m/z* = 429.1901 [M+H]^+^, calculated for [C_24_H_28_O_7_+H]^+^: 429.1908.

^1^H-NMR (499.87 MHz, DMSO-*d_6_*, 25 °C, 2.50 ppm): 0.96 (1H, td, *J* = 13.6, 3.7 Hz, H-3a), 1.01 (3H, s, H-13), 1.36 (1H, td, *J* = 13.3, 3.3 Hz, H-1a), 1.45 (1H, m, H-2a), 1.63 (1H, m, H-2b), 1.91 (1H, s,
H-5), 1.97 (1H, m, H-3b), 2.08 (3H, s, H-17), 2.11 (1H, d, *J* = 13.2, H-1b), 2.36 (1H, d, *J* = 19.0 Hz, H-7a), 2.76 (1H, ddt, *J* = 19.0, 6.0, 3.1 Hz, H-7b), 3.14 (1H, d, *J* = 10.6, H-14a), 3.61 (1H, d, *J* = 10.6 Hz, H-14b), 4.67 (1H, d, *J* = 11.0 Hz, H-15a), 4.80 (1H, d, *J* = 11.0, H-15b), 4.96 (2H, m, H-11), 5.83 (1H, d, *J* = 6.0 Hz, H-6), 7.52 (2H, m, H-4′/6′), 7.67 (1H, tt, *J* = 7.3, 1.2 Hz, H-5′), 7.97 (2H, m, H-3′/7′).

^13^C-NMR (125.70 MHz, DMSO-*d_6_*, 25 °C, 39.5 ppm): 17.3 (C-2), 20.6 (C-17), 28.2 (C-7), 30.7 (C-1), 34.6 (C-3), 39.4 (C-4), 39.8 (C-10), 52.5 (C-5), 62.9 (C-14), 65.4 (C-15), 66.5 (C-6), 71.1 (C-11), 121.2 (C-8), 128.7 (4′/6′), 129.3 (3′/7′), 129.8 (C-2′), 133.5 (5′), 165.6 (C-1′), 166.8 (C-9), 170.5 (C-16), 173.2 (C-12).

Dideacetyl parasiticolide A

HRESIMS: *m/z* = 387.1817 [M+H]^+^, calculated for [C_22_H_26_O_6_+H]^+^: 387.1802.

^1^H-NMR (499.87 MHz, DMSO-*d_6_*, 25 °C, 2.50 ppm): 0.91 (1H, ddd, *J* = 13.4, 13.4, 3.7 Hz, H-3a), 0.99 (3H, s, H-13), 1.17 (1H, dd, *J* = 12.9, 3.5 Hz, H-1a), 1.42 (1H, m, H-2a), 1.65 (1H, m, H-2b), 1.81 (1H, s, H-5), 1.97 (1H, d, *J* = 13.4 Hz, H-3b), 2.28 (1H, d, *J* = 12.9 Hz, H-1b), 2.31 (1H, d, *J* = 19.3 Hz, H-7a), 2.73 (1H, m, H-7b), 3.09 (1H, dd, *J* = 10.5, 5.3 Hz), 3.60 (1H, dd, *J* = 10.5, 5.3 Hz, H-14b), 4.07 (1H, m, H-15a), 4.12 (1H, m, H-15b), 4.42 (1H, dd, *J* = 5.3, 5.3 Hz, 14-OH), 4.86 (1H, dd,  *J* = 17.5, 2.0 Hz, H-11a), 5.06 (1H, dt, *J* = 17.5, 2.5 Hz, H-11b), 5.10 (1H, dd, *J* = 5.2, 5.2 Hz, 15-OH), 5.80 (1H, d, *J* = 5.8 Hz, H-6), 7.55 (2H, m, H-4′/6′), 7.67 (1H, m, H-5′), 7.90 (2H, m, H-3′/7′).

^13^C-NMR (125.70 MHz, DMSO-*d_6_*, 25 °C, 39.5 ppm): 17.6 (C-2), 27.3 (C-13), 28.5 (C-7), 30.5 (C-1), 34.9 (C-3), 39.4 (C-4), 42.9 (C-10), 52.7 (C-5), 62.4 (C-15), 63.1 (C-14), 66.9 (C-6), 72.0 (C-11), 119.5 (C-8), 128.9 (C-4′/6′), 129.0 (C-3′/7′), 129.8 (C-2′), 133.5 (C-5′), 165.7 (C-1′), 169.4 (C-9), 173.6 (C-12).

18-Formyl parasiticolide A

HRESIMS: *m/z* = 457.1889 [M+H]^+^, calculated for [C_25_H_28_O_8_+H]^+^: 457.1857.

^1^H-NMR (499.87 MHz, DMSO-*d_6_*, 25 °C, 2.50 ppm): 1.10 (3H, s, H-13), 1.18 (1H, ddd, *J* = 13.7, 13.7, 3.3 Hz, H-3a), 1.43 (1H, ddd, *J* = 13.2, 13.2, 2.9 Hz, H-1a), 1.51 (1H, m, H-2a), 1.71 (1H, m,
H-2b), 1.82 (1H, d, *J* = 13.7 Hz, H-3a), 2.06 (1H, s, H-5), 2.09 (3H, s, H-17), 2.16 (1H, d, *J* = 13.2 Hz, H-1b), 2.43 (1H, d, *J* = 19.3 Hz, H-7a), 2.80 (1H, m, H-7b), 3.99 (1H, d, *J* = 11.0 Hz, H-14a), 4.36 (1H, d, *J* = 11.0 Hz, H-14b), 4.71 (1H, d, *J* = 10.8 Hz, H-15a), 4.78 (1H, d, *J* = 10.8 Hz, H-15b), 4.98 (2H, m, H-11), 5.83 (1H, d, *J* = 5.6 Hz, H-6), 7.52 (2H, dd, *J* = 7.6, 7.6 Hz, H-4′/6′), 7.68 (1H, dd,
*J* = 7.6, 7.6 Hz, H-5′), 7.98 (2H, d, *J* = 7.6 Hz, H-3′/7′), 8.06 (1H, s, H-18).

^13^C-NMR (125.70 MHz, DMSO-*d_6_*, 25 °C, 39.5 ppm): 16.9 (C-2), 20.5 (C-17), 26.6 (C-13), 27.9 (C-7), 30.5 (C-1), 35.4 (C-3), 36.9 (C-4), 39.6 (C-10), 52.1 (C-5), 65.0 (C-14), 65.1 (C-15), 66.5 (C-6), 71.1 (C-11), 121.0 (C-8), 128.6 (C-4′/6′), 129.0 (C-3′/7′), 129.2 (C-2′), 133.4 (C-5′), 161.6 (C-18), 165.1 (C-1′), 165.9 (C-9), 170.1 (C-16), 172.8 (C-12).

Ditryptoleucine A

HRESIMS: *m/z* = 625.3472 [M+H]^+^, calculated for [C_36_H_44_N_6_O_4_+H]^+^: 625.3497.

^1^H-NMR (499.87 MHz, DMSO-*d_6_*, 25 °C, 2.50 ppm): 0.81 (6H, d, *J* = 6.4 Hz, H-21), 0.83 (6H, d,  *J* = 6.4 Hz, H-20), 1.42 (H2, m, H-18a), 1.48 (H2, m, H-18b), 1.58 (H2, m, H-19), 2.44 (H4, m, H-11), 2.80 (H6, s, H-17), 3.76 (H2, dd, *J* = 10.4, 3.8 Hz, H-12), 3.81 (H2, dd, *J* = 7.9, 6.0 Hz, H-15), 5.09 (H2, m, H-2), 6.61 (H2, d, *J* = 7.8, H-5), 6.66 (H2, t, *J* = 7.5, H-7), 6.68 (H2, s, H-3), 7.05 (H2, t, 7.8, H-6), 7.23 (H2, d, *J* = 7.8, H-8).

^13^C-NMR (125.70 MHz, DMSO-*d_6_*, 25 °C, 39.5 ppm): 21.9 (C-20), 22.6 (C-21), 23.7 (C-19), 31.8 (C-17), 36.1 (C-11), 39.4 (C-18), 56.7 (C-12), 57.9 (C-10), 61.6 (C-15), 76.7 (C-2), 108.7 (C-5), 117.2 (C-7), 124.6 (C-8), 126.7 (C-9), 129.0 (C-6), 150.9 (C-4), 165.0 (C-16), 166.1 (C-13).

Ditryptoleucine B

^1^H-NMR (499.87 MHz, DMSO-*d_6_*, 25 °C, 2.50 ppm): 0.81 (6H, d, *J* = 6.1 Hz, H-21), 0.83 (6H, d,  *J* = 6.2 Hz, H-20), 1.42 (H2, m, H-18), 1.49 (H2, m, H-19), 2.45 (H2, dd, *J* = 13.5, 4.7, H-11a), 2.75 (H6, s, H-17), 3.13 (H2, m, H-11b), 3.33 (H2, s, H-2), 3.82 (H2, t, *J* = 6.9 Hz, H-15), 4.23 (H2, m,
H-12), 6.58 (H2, d, *J* = 7.5, H-5), 6.65 (H2, t, *J* = 7.1, H-7), 6.70 (H2, s, H-3), 7.01 (H2, t, 7.2, H-6), 7.37 (H2, d, *J* = 7.1, H-8).

^13^C-NMR (125.70 MHz, DMSO-*d_6_*, 25 °C, 39.5 ppm): 21.5 (C-21), 22.5 (C-20), 23.6 (C-19), 31.6 (C-17), 37.0 (C-11), 38.0 (C-18), 55.3 (C-12), 60.8 (C-10), 61.7 (C-15), 76.3 (C-2), 108.5 (C-5), 117.4 (C-7), 124.0 (C-8), 130.2 (C-9), 128.3 (C-6), 149.6 (C-4) 166.9 (C-16), 166.6 (C-13).

Oryzamide A_1_

HRESIMS: *m/z* = 491.26526 [M+H]^+^, calculated for [C_28_H_34_ N_4_O_4_+H]^+^: 491.26525.

^1^H-NMR (499.87 MHz, DMSO-*d_6_*, 25 °C, 2.50 ppm): 0.83 (3H, d, *J* = 6.5 Hz, H-5′), 0.88 (3H, d,  *J* = 6.6 J, H-5′), 1.36 (1H, m, H-4′), 1.59 (2H, m, H-3′), 1.77 (3H, s, H-2′′′), 2.67 (1H, m, H-3′′), 2.83 (1H, m, H-3′′), 2.87 (3H, s, N-CH), 4.76 (1H, m, H-2′′), 5.02 (1H, m, H-2′), 6.47 (1H, d, *J* = 14.8,
H-2), 6.61 (2H, d, *J* = 8.4 Hz, H-6′′/8′′), 7.01 (2H, d, *J* = 8.4 Hz, H-5′′/9′′), 7.05 (1H, m, H-8), 7.10 (1H, m, H-7), 7.26 (1H, m, H-1), 7.36 (1H, m, H-6), 7.41 (1H, s, H-4), 7.62 (1H, d, *J* = 7.9 Hz, H-9), 8.24 (1H, d, *J* = 8.0 Hz, 2′′-NH), 9.16 (1H, s, 7′′-OH), 9.84 (1H, d, *J* = 9.8 Hz, 1-NH), 11.11 (1H, s, 4-NH).

^13^C-NMR (125.70 MHz, DMSO-*d_6_*, 25 °C, 39.5 ppm): 21.6 (C-5′), 22.0 (C-2′′′), 22.7 (C-5′), 24.1 (C-4′), 30.9 (C-N), 36.2 (C-3′′), 36.9 (C-3′), 50.5 (C-2′′), 54.2 (C-2′), 106.4 (C-2), 111.4 (C-3), 111.5 (C-6),114.6 (C-6′′/8′′), 118.5 (C-9), 118.8 (C-8), 119.2 (C-1), 121.1 (C-7), 123.1 (C-4), 125.0 (C-10), 127.4 (C-4′′), 129.7 (C-5′′/9′′), 136.9 (C-5), 156.0 (C-7′′), 167.7 (C-1′), 168.8 (C-1′′′), 172.3 (C-1′′).
